# Synergetic Benefits of Agricultural Sewage Reuse and Floating Photovoltaics in Mexican Wastewater System: A Municipal‐Level WEF Nexus Study

**DOI:** 10.1002/gch2.202500440

**Published:** 2026-01-14

**Authors:** Shahin Rasooli, César Casiano‐Flores, Shahrzad Farhoodi, Bassel Daher, Pabel Antonio Cervantes‐Avilés, Carlos Alberto Huerta‐Aguilar

**Affiliations:** ^1^ School of Engineering and Sciences Tecnologico de Monterrey Puebla Mexico; ^2^ Section of Governance and Technology for Sustainability University of Twente Enschede Netherlands; ^3^ Department of Biological and Agricultural Engineering Texas A&M University College Station USA

**Keywords:** floating photovoltaics, river remediation, techno‐economic assessment, wastewater reuse, WEF Nexus

## Abstract

Financial stability is essential for the sustainable operation of wastewater treatment plants (WWTPs), as they require substantial resources but generate minimal revenue. In Mexico, a lack of funding resources and supporting policies has resulted in inefficient treatment and extensive surface water degradation. This work examines the positive impacts of treated sewage‐operated irrigation ponds equipped with floating photovoltaics on the WWTP's economic viability in central Mexico. These ponds will provide water for irrigation expansion in rainfed cornlands, and the harvested solar energy will be exported to the national grid to independently generate income. The turnover will cover capital and operational expenditures of WWTPs, irrigation ponds, FPV, and energy for irrigation. The proposal's performance is evaluated through techno‐economic and Water‐Energy‐Food Nexus assessments on municipal level. It is projected that by in‐situ recycling 35% of generated sewage, local corn growers could benefit from an additional 45 320 hectares (223%) of irrigated cornland, over 5 46 000 tons (132%) of corn grain production, and 139% more sales revenue. The overall power capacity of FPV units could reach 834 MW, and 1796 GWh of clean energy could be harvested annually. This example demonstrates the value proposition of irrigation ponds and FPVs on the sustainability of existing WWTPs globally.

## Graphical abstract

This study proposes integrating treated sewage irrigation ponds with floating photovoltaics to enhance wastewater treatment plants’ financial viability in the Atoyac River Basin, central Mexico. The model demonstrates potential to strengthen the water‐energy‐food nexus by recycling one‐third of sewage, increasing irrigation acreage on cornlands, boosting yields, generating clean energy, and securing the financial sustainability of the wastewater sector.

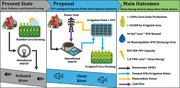

## 1 Introduction

Population growth and changing lifestyles are the main drivers of resource overexploitation, resulting in significant waste generation [1]. The growing scale of waste generation, especially wastewater, highlights the importance of the techno‐economic viability of the wastewater treatment process. On the demand side, agriculture represents the largest footprint on global freshwater abstraction, while wastewater reuse under stringent treatment conditions can ease irrigation water demand [2] and enhance farm economics through nutrient recovery [3]. Any failure in the sustainable operation of wastewater treatment plants (WWTPs) can lead to environmental degradation. For instance, in Mexico, insufficient funding for OPEX has resulted in nationwide surface water pollution, where the governance structure is a key contributor to this flawed mechanism [4]. Under this scheme, the initial investment and installation of WWTPs are mostly led by the federal government, and OPEX is funded by municipalities with the support of state governments [5]. The lack of financial self‐sufficiency (access to the budget) in WWTP's management has resulted in other expenses being prioritized by subnational governments [6]. The outcome of these dynamics is widespread abandonment and deterioration of treatment facilities nationwide [7]. As a critical case, the Atoyac River basin is now a highly contaminated river system due to large volumes of untreated or poorly treated discharges, negatively affecting the livelihoods of approximately 5.4 million people [8] in central Mexico [9].

Regardless of wastewater management's contribution to public health and its capacity to partially meet the demand of the largest water consumer, it represents an unsustainable business model for generating income. As a result, the required capital expenditures (CAPEX) and operational expenditures (OPEX) are conventionally covered through governmental budgets [10, 11]. To directly generate income from the wastewater treatment process, auxiliary technologies such as biogas [12] or hydrogen [13] production are often proposed. However, additional conversion packages are also required to harvest heat and/or power from the gaseous streams generated. Accordingly, the imbalance between cost and revenue often discourages the integration of such processes, which explains why these techniques are rarely used as an income source for WWTPs. In this regard, proposing new viable and standalone approaches could be key to addressing the challenges of monetizing the wastewater treatment process.

As a trending renewable energy technology (RET) within the water–energy nexus, floating photovoltaics (FPVs) have gained global traction in recent years [14], and their performance has been evaluated from multiple perspectives. Their proximity to the water surface enhances panel cooling and typically yields higher performance than conventional ground‐mounted photovoltaics (GPV) [15]. Experimental studies also have shown that the overall yields can be further increased by integrating optimization techniques into the DC‐AC conversion in the inverters [16]. From a techno‐economic viewpoint, FPV systems have also demonstrated their viability and success across different geographical contexts (e.g., India [17], Egypt [15]) and deployment settings (natural [18] and artificial [19, 20] water bodies), confirming their reliability. In particular, recent developments and cost reduction in photovoltaics (PV) [21] make this technology a promising option for integration with WWTPs.

Beyond the Mexican wastewater sector, the agricultural and rural systems are also facing significant challenges. While the market access created by the North American Free Trade Agreement (NAFTA) has provided new opportunities for northern and northwestern states [22], the central and southern regions still struggle with rainfed corn farming, mainly for personal use and local domestic markets [23]. To date, the agricultural system has shown resistance to development strategies because of the Ejido landownership model, and many modernization efforts have failed due to factors such as small landholding [24] and weakly managed funding programs [25]. As a result, rural communities remain vulnerable, farming practices are often substandard, and government support is frequently misused, with little real impact [26].

On the whole, this study aims to develop a practical solution to address the aforementioned challenges. To that end, a circular economy model has been developed that couples FPV–equipped irrigation ponds with WWTPs. The harvested energy generates income through export to the grid, and the stored water provides a sufficient supply to expand irrigation. Despite advances in both agricultural wastewater reuse and FPV research, this work contributes to the literature in four main ways. First, it amplifies the availability of relatively small sewage flows to fulfill large irrigation demand, an effect often overlooked by models that rely on annual water balances [2, 27]. Second, providing a direct and in‐situ revenue stream with minimal executional challenges for WWTPs. Third, estimating corn yield improvements under expanded irrigation to quantify the agricultural benefits of the proposed model. Fourth, offering a river‐remediation approach that can instantaneously reduce influent pollutant loads in contrast to long‐term strategies. To evaluate this concept, we applied a comprehensive WEFN and techno‐economic analysis on the municipality level for the Atoyac River basin.

## 2 Materials and Methods

### 2.1 Study Area

**FIGURE 1:**
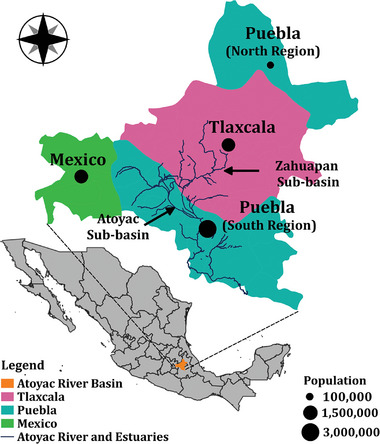
The geographical outline and population of the Atoyac River basin's municipalities and corresponding states in Central Mexico. Mexico State stands for the western municipalities; Puebla for northern and southern municipalities, and, due to the geographical landscape, Tlaxcala is surrounded by Puebla from north, south, and east. The figure was developed by the authors according to the municipalities of the basin as reported in [32].

The region studied in this paper is the Upper Atoyac River basin (in Spanish, *Alto Rio Atoyac*), a tributary of the Balsas River in the Central‐South region of Mexico. The Atoyac River basin lies within the Tlaxcala‐Puebla Valley, a volcanic and mountainous zone bordered by Malinche, Iztaccíhuatl, and Popocatepetl mountains [9]. The basin is in the eastern zone of Mexico City, the country's capital, and encompasses eighty‐two municipalities from the States of Tlaxcala (52), Puebla (26), and Mexico (4) with a total population of 5.4 million inhabitants. Socially, all three states are categorized as highly developed states in terms of the Human Development Index (HDI > 0.7) [28]. Since the implementation of industrial decentralization policies in Mexico City, neighboring states have received more industrial investments [29]. Nowadays, economic activities are based on manufacturing, automotive, petrochemicals, pharmaceuticals, agriculture, and textile [30]. Due to the adequate climate of the region and fertile soil texture, agroindustry occupies over 3 00 000 hectares of land for grains, perennials, and vegetable production, whereas corn, with approximately 1 72 000 hectares of acreage, is the major cultivated crop [31]. Figure 1 illustrates the spatial distribution of the Alto Atoyac River basin within the Puebla‐Tlaxcala valley. After Puebla, the Atoyac River merges with the Balsas River, flowing through Morelos and Guerrero states, before reaching the Pacific Ocean.

### 2.2 Data Sources

At the municipality level, agrometeorological data have been obtained from the Fishing and Agriculture Information Service (Servicio de Información agroalimentaria y Pesquera, SIAP) database (e.g., cultivated area, type of crops, irrigation pattern, market price, and land productivity), and meteorological data have been obtained through Meteonorm V8 software based on municipality coordinates. Municipality population and water consumption patterns have been obtained from the National Institute of Statistics and Geography (Instituto Nacional de Estadística y Geografía, INEGI) database and Rapid Evaluation of Energy Use (Evaluación Rápida del Uso de la Energía) official report for Puebla State. The crop planting calendar is developed for central Mexico's climate (Section S1). Also, sewage characteristics and required treatment performance for safe irrigation were obtained from FAO [33] and NOM‐001‐SEMARNAT‐2021 [34] (Section S2).

### 2.3 Revenue Mechanism and Irrigation Expansion Strategy

**FIGURE 2:**
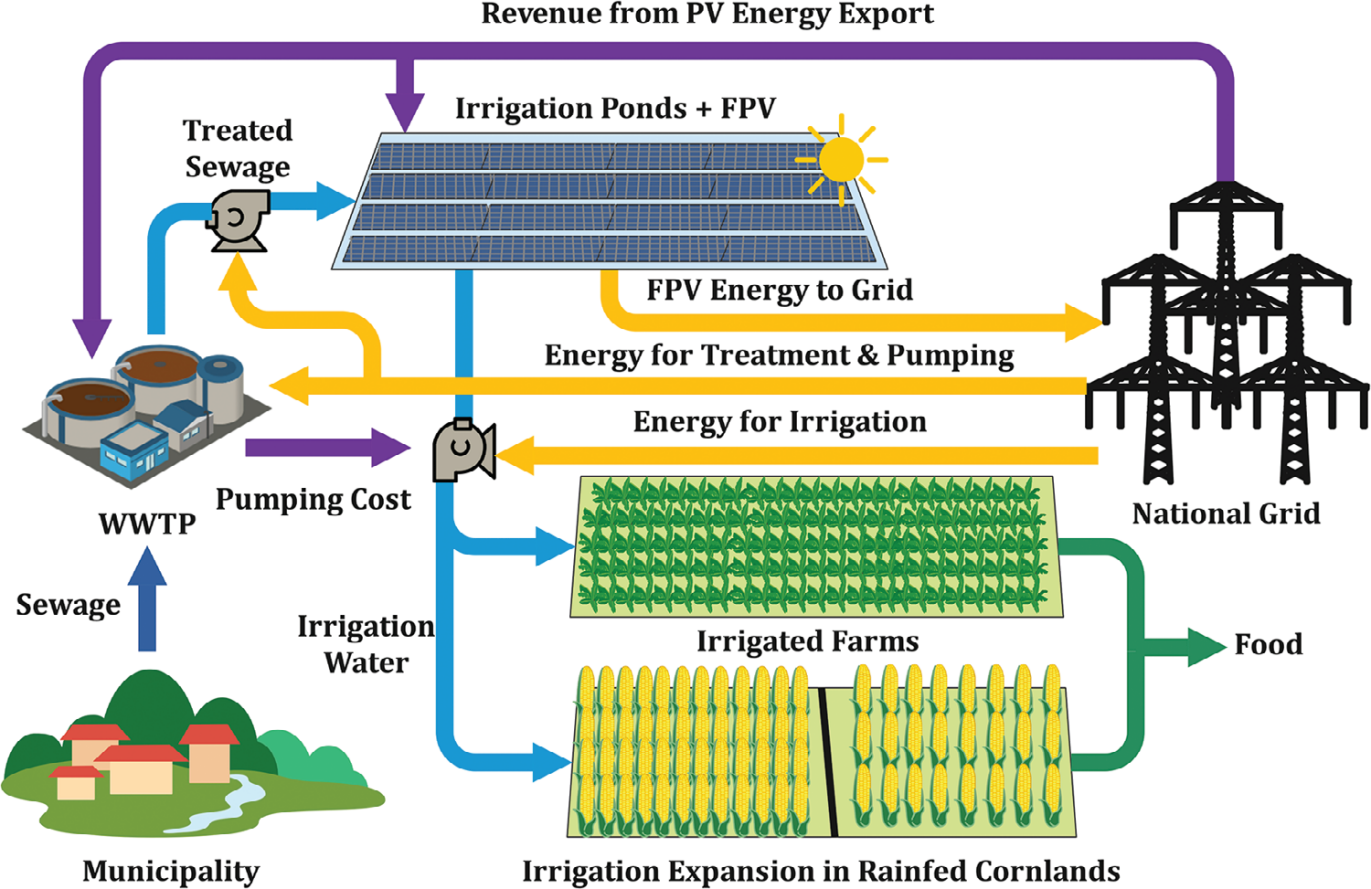
The schematics of the proposed revenue mechanism for WWTPs and irrigation expansion by treated sewage in the Atoyac River basin. The figure was developed by the authors.

Figure 2 illustrates the proposed revenue mechanism and sewage‐driven irrigation expansion to reduce sewage discharges by viable WWTP operation. The generated sewage from municipalities is collected and treated in decentralized WWTPs, then transferred to coupled irrigation ponds where FPVs are installed to save water from evaporation. The built water inventory will provide sufficient water for irrigation expansion in rainfed cornlands as well as existing irrigated farms. The photovoltaic (PV) energy generated from the FPV units will be exported to the grid, and the generated revenue will be used to cover OPEX of WWTP, irrigation ponds, and pumping cost for running the irrigation system, while for FPV units, both CAPEX and OPEX will be compensated.

### 2.4 Irrigation Water Availability, Demand, and Productivity

Irrigation water availability is calculated according to per capita water consumption (0.172 m^3^·capita^−1^·day^−1^) and sewage generation rate (80%), which has been reported by local authorities [35]. On the demand side, according to the meteorological and agricultural statistics, irrigation water demand for each municipality has been estimated. Theoretical water demand is calculated by the Penman‐Monteith equation (Equation (1)) developed by the Food and Agriculture Organization (FAO), using a reference crop evapotranspiration (ET_0_) [36]. The estimated ET_0_ is used for other crops by multiplying to the crop coefficient (K_c_) (Equation (2)) [36]. Under actual conditions, crop evapotranspiration could be different than theoretical values, hence water stress coefficient is also introduced. For water demand, CROPWAT 8.0 model developed by FAO has been applied for corn and currently irrigated crop species [37]. It is worth noting that we considered a 20% water loss in the irrigation system's operation [2]. We developed Equation (3) to estimate the irrigation demand according to the monthly available precipitation.

(1) $$ET_{0}=\frac{0.408\times \delta \times \left(R_{n}-G\right)+\gamma \times \frac{900}{T+273}\times U_{2}\times \left(e_{s}-e_{a}\right)}{\delta +\gamma \times \left(1+0.34U_{2}\right)}$$

(2) $$ET_{a}=K_{s}\times K_{c}\times ET_{0}$$

(3) $$\mathrm{ID}=(ET_{a}-\frac{\mathrm{ER}}{3})\times L\times C$$

where *ET_0_* and *ET_a_* are reference and actual crop evapotranspiration (mm·10‐day^−1^); *R_n_* is net solar radiation on the crop surface (MJ·m^−2^·day^−1^); *G* is soil heat flux (MJ·m^−2^·day^−1^); *T* is average air temperature (°C); *U_2_* is the wind speed at 2 m above the ground (m·s^−1^); *e_s_* and *e_a_* are saturation and measured vapor pressure respectively (kPa); δ is the saturation vapor pressure vs. temperature curves slope (kPa·°C^−1^); γ is the hydrometer constant (kPa·°C^−1^); K_C_ is crop evapotranspiration coefficient compared to reference crop; K_S_ represents the water stress coefficient which is often assumed as 1 for water demand calculations; ID is the farm's irrigation water demand calculated (m^3^·day^−1^); *ER* is the monthly effective rainfall (mm·month^−1^); *L* is the planting landscape (ha); C is the unit conversion factor that based on respective values of ID_,_ *ET*, and *L* is equal to 1. It is worth noting that the ID value for each crop type is generated by CROPWAT for 10‐day intervals, considering rainfall profiles (mm·10‐day^−1^). Irrigation water demand is a time‐dependent value, hence, for each crop, the time‐based irrigation water demand has been transformed to a pulse function by Heaviside transformation [38]. This approach helps to have a realistic estimation of the pond water level and capacity, and avoid unnecessary pond size.

Crop water productivity functions are applied to predict on‐farm productivity. As shown in Equation (4), crop water productivity is a polynomial equation in which coefficients are identified by regression, and Y is the crop yield per unit area of land (ton·ha^−1^). In this regard, the FAO's AquaCrop model has been applied to estimate the grain yields of cornlands for each municipality [39]. This model requires climate profiles including *ET_0_*, temperature profiles, rainfall patterns, and CO_2_ availability, as well as crop models to evaluate farm performance through specified planting dates. According to the water availability, this model provides the farm response to proper irrigation.

(4) $$Y=a\times {\mathrm{ET}}_{a}^{2}+\;b\times ET_{a}+\;c$$

(5) $$ET_{a}=\min\left(-\frac{b}{2a}\pm \sqrt{\frac{Y}{a}+\frac{b^{2}}{4a^{2}}-\frac{c}{a}}\right)$$

### 2.5 Energy Demand and Harvesting in Wastewater Management

Interlinkages between water and energy are based on the energy requirement for wastewater treatment, pond operation, water transfer from pond to farm, and available area for floating photovoltaic installations. Regarding the final irrigation demand for the municipalities with extra sewage resources, the daily treatment capacity is calculated according to the irrigation expansion capability. The energy requirement for sewage collection and transfer from households to treatment points is assumed to be zero, as these systems rely on gravity flow. The required energy for treatment is calculated considering the amount of annually treated sewage and the selected treatment technology.

Based on the maximum water level during a year, the maximum capacity of the ponds has been estimated. A square reservoir configuration with a 3‐m depth has been assumed to have the water surface area for solar panel implementation. The mathematical equations of water balance in the pond system are shown below. The pond volume level equation was resolved by MATLAB R2024a, Ordinary Differential Equations (ODE) toolbox [40]. An accurate determination of irrigation pond size requires a two‐step ODE model resolution. Initially, the model is solved by assuming a zero initial volume within the pond. Subsequently, the minimum water level was applied as the starting volume for the second attempt (Equation (6)). This approach facilitates the calculation of a realistic pond size based on the specific irrigation water demands and established watering schedules of individual municipalities. According to the maximum water level in the pond system and the required number of WWTPs, the individual pond capacity and total area have been calculated.

(6) $$\begin{array}{c} d\frac{V_{j}}{\mathrm{dt}}=\mathrm{TS}I_{j}-\mathrm{IW}H_{j}\left(t\right)\\ i.c\;1:V_{j}\left(t=0\right)=0\\ i.c\;2:V_{j}\left(t=0\right)=\min\left(\mathrm{abs}\left(\mathrm{Vj}\right)\right) \end{array}$$

where V_j_ is the water level in the irrigation pond system for municipality j (m^3^); TSI_j_ is the daily treated sewage influent value (m^3^·day^−1^); IWH_j_(t) is the irrigation water demand's Heaviside function (Section S3) for municipality j (m^3^·day^−1^). Also, from pond to farm, the required energy is assumed to be 0.190 kWh m^−3^. The installed irrigation ponds are assumed to be utilized as places to implement floating FPV systems. The required climatological data for FPV modeling were retrieved from the PVGIS portal [41]. First, it is necessary to quantify the water surface's cooling effect on solar panel temperature. For this reason, we used the modified Faiman's cell temperature [42] equation (Equation (7)) as implemented in the PVlib Python library [43].

(7) $$T_{c}=T_{a}+\frac{\alpha \;\times E\times \left(1-\eta_{m}\right)}{u_{c}+u_{v}\times WS}$$

where *T_c_* and *T_a_* are cell and ambient temperature (°C or K); α is the absorbed fraction of the incident irradiance and here set at 0.9; *E* is the plane‐of‐array (POA) irradiance on a given tilted surface in (W·m^−2^) and retrieved on an hourly basis from PVGIS considering optimum tilt and the azimuth of 23° and −21°. The direct component of the POA irradiance was adjusted for incidence angle effects using the Martin‐Ruiz incidence angle modifier (IAM) model [44]; *u_c_* and *u_v_* are wind‐independent and wind‐dependent heat loss coefficients, which are set at 31.9 and 1.5 [45], respectively (w·m^−2^·K^−1^); WS is the wind speed in (m·s^−1^), for the height at which heat loss coefficients are estimated. Since the obtained wind speed values from PVGIS are for a 10 m height, we used Equation (8) to estimate the wind speed values at a 2 m height, representing the wind speed exposure at the module's surface [46].

(8) $$WS_{2}=WS_{10}\times \frac{\ln\left(\frac{2}{z_{0}}\right)}{\ln\left(\frac{10}{z_{0}}\right)}$$

where *WS*_2_ and *WS*_10_ are wind speed values at 2 and 10 m heights, respectively, and *z*_0_ is the water surface roughness set to 0.001 (m) [47]. The obtained POA solar irradiance and calculated solar panel temperature are used to estimate the power output of a commercial "ET‐M772BH550WW/WB 550W" panel, which has a maximum DC capacity of 550 W and a temperature coefficient of −0.0034 (°C^−1^). To that end, we employed the National Renewable Energy Laboratory's (NREL) PVWatts model in the PVlib Python library to estimate the DC and AC outputs of a single panel coupled with a 600 Wac inverter, assuming reference and nominal efficiencies of 0.963 and 0.970, respectively. The hourly AC power outputs were then aggregated and scaled according to the number of panels in each irrigation pond's FPV system. The technical details of the panel's geometry, specification, and system installation are presented in Section S4.

### 2.6 Techno‐Economic Assessment

The techno‐economic assessment covers the cost and revenue estimations of FPV units, irrigation ponds, and WWTPs. The studied economic evaluation metrics are Farmer Revenue Change (FRC), Net Present Value (NPV), and Implicit Water Value (IWV). First, CAPEX and OPEX calculations for the FPV systems are obtained from the NREL report, where we selected a 2 MW capacity as the basis of calculations [48]. The power purchase rate of the harvested solar energy is set to 107.75 USD·MWh^−1^ [49] equal to the household electricity price in 2023, since net metering for harvested solar energy is applied for photovoltaic energy sales. Land cost for irrigation ponds is set at 50000 USD·ha^−1^. Additionally, we carried out a sensitivity analysis to demonstrate the maximum and minimum thresholds of purchase rate to meet economic feasibility (positive NPV) to afford the most cost‐effective and expensive treatment technologies.

For the techno‐economic assessment of WWTPs, we considered four treatment technologies and pond systems that address the safety regulation of treated sewage irrigation. The OPEX and process descriptions of these technologies are presented in Section S5. The sizing of the WWTPs is considered equal to 2000 p.e. (people equivalent) capacity to address decentralized and small‐scale treatment unit criteria [50]. To have an accurate cost estimation, cost results are updated and tailored for Mexico and 2022 using cost indexing. We used the indices reported by the Process Economics Program Cost Index (PCI#107) published by “S&P Global” [51]. The US Gulf Coast (USGC) and Germany, as representatives of Western Europe, have been selected as original location factors for FPV and WWTPs, respectively, while South America, as a representative of Mexico [52]. The supplementary information annex (Section S6) presents the cost updating and formulation details.

To evaluate the economic performance of this study, we used FRC (%), NPV (USD), and IWV (USD·m^−3^) as economic metrics. FRC is calculated based on the projected revenue after irrigation expansion (higher yields in irrigated and transformed rainfed cornlands according to AquaCrop results) and compared to current turnovers. The cost side of NPV includes the yearly OPEX of FPV and WWTPs, the cost of the energy required to operate the irrigation system, and the Total Annualized Equivalent Cost (TAEC) of FPV and the irrigation pond's land cost, as calculated using Equation (9) [50]. Revenue is generated through solar power sales to the Mexican national grid, and finally, NPV is calculated using Equation (10) [50].

(9)

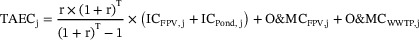

(10) $$\mathrm{NP}V_{j}=\sum_{t=0}^{T}\frac{{\left(R_{j}-\left(\mathrm{TAE}C_{j}+\mathrm{IE}C_{j}\right)\;\right)}_{t}}{{\left(1+r\right)}^{t}}$$

where TAEC_j_ is the total annualized equivalent cost in municipality j (USD·year^−1^); IC is the initial capital investment for CAPEX of FPVs and annual depreciation of land cost (USD); r is the discount rate, here average interest rate of previous large‐scale PV units in Mexico [53] (6.4%) is used; T is the lifetime of the Project which is assumed to be 25 years; NPV_j_ is the net present value of the project (USD); t is the year counter; R_j_ is revenue of the FPV units by energy sales to the grid (USD·year^−1^); O&MC is the operation and maintenance cost (equivalent to OPEX) of FPV and WWTPs (USD·year^−1^); IEC_j_ is the cost of energy for running the irrigation system (USD·year^−1^). Finally, IWV is calculated by the annual cashflow, (the numerator of Equation (10)) of the system per annual treated sewage amount of the municipality (USD·m^−3^).

## 3 Results

Details of the model's results for each municipality are presented in the Data S1. In the following subsections, the obtained results on the basin level are reported.

### 3.1 Water and Agriculture

**FIGURE 3:**
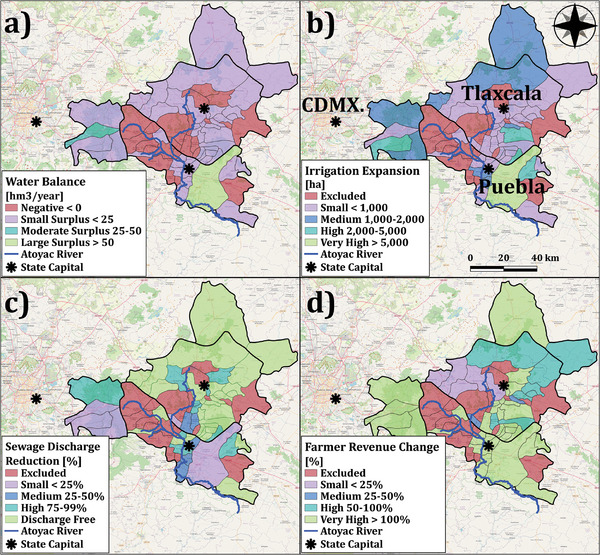
(a) Water Balance between sewage availability and irrigation water demand [hm^3^·year^−1^], (b) Expanded irrigation in rainfed cornlands [ha], (c) Sewage discharge reduction [%], (d) Farmer revenue change [%]. The figure was developed by the authors according to the results.

Figure 3a demonstrates the spatial distribution of the water balance in the basin municipalities, considering the annually available sewage resources and required freshwater for irrigation. The total annual water requirement for domestic applications and corresponding sewage generation amounts is estimated to be 335 and 268 hm^3^, respectively. On the demand side, current irrigated farmlands require 106 hm^3^ of freshwater annually in addition to precipitation. While the total annually available sewage surpasses the required water for irrigation, the geographically distributed nature of the water balance creates inequalities. Therefore, municipalities with positive balances were selected to implement this proposal, and 27 municipalities were excluded due to a lack of 34 hm^3^·year^−1^ of sewage to fulfill the irrigation demand.

Municipalities with sufficient treated sewage are included for irrigation expansion and system upgrading; thus, 55 municipalities are engaged by the model. Consequently, irrigation practices could be transformed into 45 320 hectares of rainfed cornlands (Figure 3b). In this regard, 94 hm^3^·year^−1^ of treated sewage could be redirected towards current irrigated farms and transformed rainfed cornlands. Specifically, 63 hm^3^ will be devoted to corn irrigation, with water productivity in corn grain production estimated at 10.8 kg m^−3^ (6 78 509 ton of grain would be harvested from cornlands by standard irrigation). Sewage recovery and reuse by 35% could remove the footprint of 36 municipalities from sewage discharge in the Zahuapan River's estuaries. However, the metropolitan area of Puebla City and densely populated municipalities in the southern and western zones would still contribute to the largest discharges. Figure 3c demonstrates the potential sewage discharge reduction after irrigation expansion.

**FIGURE 4:**
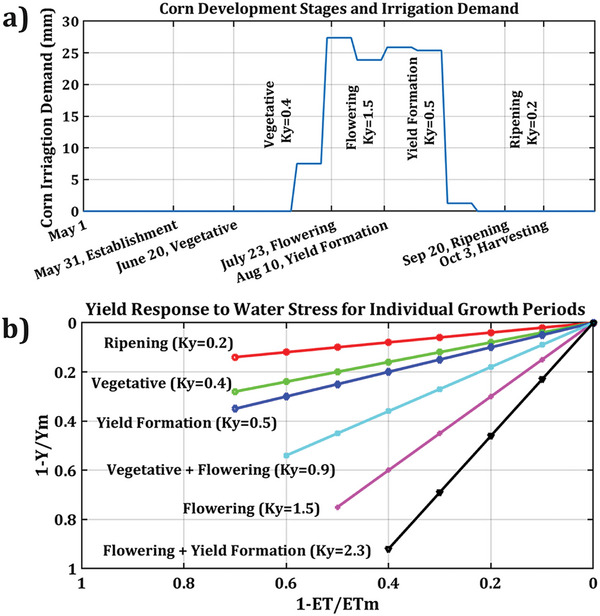
Relationship between corn growth stages, irrigation needs, and yield response to water stress across development phases. a) Corn development stages and irrigation demand based on the agricultural calendar, b) Relative corn grain yield response to water stress for growth stages (FAO‐Maize). ET and ETm are actual and maximum evapotranspiration. Y and Ym are the actual and maximum yield of the crop. (a) has been developed by the authors using the irrigation water demand estimation; (b) has been redrawn using the data retrieved from [54].

From an agricultural viewpoint, standard and safe irrigation with treated sewage can boost crop yield and farm economic productivity. Corn is biologically sensitive to water stress (FAO‐Maize). Figure 4a illustrates the required water for irrigation in the basin peaks between late July and early September (approximately 60 days), coinciding with the flowering and yield formation stages of plant growth. During this period, corn's response to water stress significantly increases yield loss by a factor of 2.3 [54] (Flowering + Yield Formation line's slope in Figure 4b), highlighting its unsuitability for a rainfed regime in the basin's settings (Figure 4b). Expanding irrigation using recovered sewage water, instead of discharging it into the Atoyac River, can improve corn yields and support environmental conservation. In 2022, the average yield and total corn grain production were 2.4 ton·ha^−1^ and 4 14 040 ton, respectively. With irrigation expansion, it is projected to achieve yields of 5.6 ton·ha^−1^ and a total production of 9 60 310 ton. The average yield with irrigation will increase from 4.4 to 11.2 ton·ha^−1^. Consequently, standard irrigation will contribute to 71% of total corn grain production in the basin, even though only 32% of cornlands would be equipped with standard irrigation systems. According to local corn grain prices in 2022 and projected production capacity, the agroindustrial sector's revenue from corn farming can increase from 128 to 305 million USD, showing a promising 139% improvement. Comparatively, basin farms’ economic yield could sharply rise from 773 to 1776 USD/ha, positioning the basin above the national average of 1722 USD/ha. Figure 3d geographically depicts the farmer's revenue change in the basin municipalities.

### 3.2 Techno‐Economic Assessment

**FIGURE 5:**
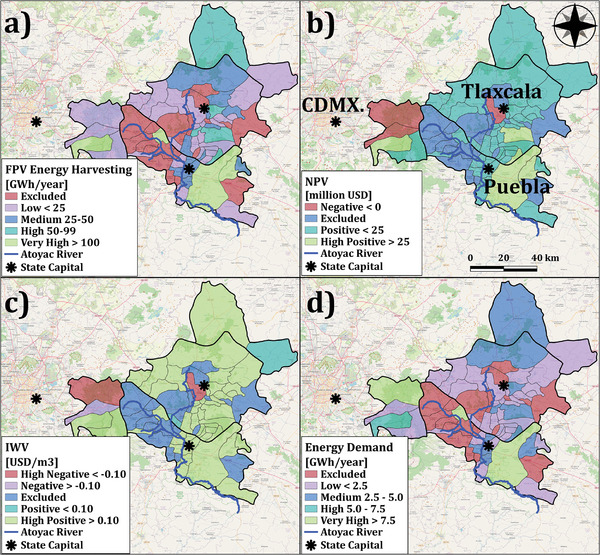
(a) FPV energy harvesting [GWh·year^−1^], (b) Net Present Value [million USD], (c) Implicit Water Value (IWV) [USD·m^−3^], (d) Energy Demand [GWh·year^−1^]. The figure was developed by the authors according to the results.

The pond surface area is calculated based on the maximum water level, determined by the irrigated farmland acreage, crop types, and cornland irrigation demand. The total pond surface area will be 624 ha, averaging 1.3hectares per treatment unit, and will support irrigation for 45 320 ha of rainfed cornlands, significantly boosting agricultural productivity. Individual pond sizes vary across the basin, with a range from 0.2 to 1.8 hectares per treatment unit. The estimated installation capacity of FPV is 834 MW, with an annual harvestable energy potential of 1796 GWh, according to the outputs of the developed Python model and retrieved data from PVGIS. The total required CAPEX is projected at 1349 million USD, with annual OPEX for maintenance and running expenses estimated at 16 million USD. The proposed FPV configuration could generate approximately 194 million USD annually by exporting solar energy to the Mexican national grid, which will offset the OPEX of WWTPs, energy costs for irrigation systems’ operation, and FPV systems’ OPEX. Figure 5a depicts the geographical distribution of FPV systems’ solar energy yield. It is worth noting that the projected investment per megawatt of power capacity for FPV systems in the Atoyac River Basin is approximately 1.6 million USD (FPV CAPEX and land cost) in 2022, comparable to the 1.4 million USD invested in 2018 for the country's largest land‐based PV systems in Villanueva, Northern Mexico [53]. However, technological developments in the PV sector have reduced the investment cost globally and highlighted the importance of updated cost benchmarks [21].

To evaluate the economic feasibility of installing irrigation ponds and FPV units, we used NPV as the main measure. The cost model includes OPEX for FPV and WWTP, the land cost for irrigation ponds, and the energy cost for running the irrigation system. Revenue comes from selling solar energy to the national grid. Basin‐wide, the energy cost for the irrigation system and land's TAEC values would be 1.9 and 2.5 million USD annually, plus 15.8 million USD of OPEX for FPV. The OPEX for WWTPs varies based on the treatment technology chosen. To maximize NPV, we selected Rotating Biological Contactor (RBC) technology as the most cost‐effective choice, with an annual OPEX of 25 million USD across 491 units. RBC technology uses a thin biofilm of aerobic microorganisms on slowly rotating discs to remove organic pollutants from wastewater. While half of each disc is submerged, allowing micro‐organisms to feed on organic matter, the other half is exposed to air to boost aerobic degradation [55]. This cost‐revenue balance will generate 41 million USD annually, resulting in an NPV of 507 million USD over 25 years with a 6.4% interest rate. On a municipal level, 52 out of 55 model‐engaged municipalities demonstrate the economic feasibility of installing FPV and irrigation pond systems, with a cumulative NPV of 527 million USD. Figure 5b shows the distribution of NPV across the basin, where municipalities with unbalanced FPV energy yields and sewage management costs have not achieved economic profitability. The power purchase rate agreement is the centerpiece for the economic feasibility of this model. For NPV calculations, a fixed price scenario of 108 USD·MWh^−1^ was used [49]. A sensitivity analysis was also conducted to determine the maximum and minimum thresholds of solar energy sales prices. It was concluded that municipalities with larger treatment and pond capacities could tolerate lower energy purchase rates due to higher potential solar energy yields. Overall, the maximum and minimum power purchase rates range from 90.4 to 332.6 and 78.5 to 185.8 USD·MWh^−1^, respectively.

In Mexico, water for agricultural purposes is subsidized due to concerns about the well‐being of farming communities. Integrating solar energy development and proper water management could indirectly improve water valuation. According to the projected cash flows across municipalities, solar harvesting from pond surfaces can generate −0.2 up to 0.8 USD·m^−3^ for the stored water. It is worth noting that the current irrigation water supplied by the government has a multifactor pricing scenario according to local water availability, the source of water, and required energy for abstraction and delivery to farm settings [56]. Figure 5c shows the geographical distribution of IWV, where municipalities with higher treatment capacity and irrigation water demand would benefit from higher IWVs due to larger pond sizes. To cast light on the nexus between water and energy, the energy demand to cover treatment, storage, and pumping from the pond to farms has been calculated. Due to the selected technology for treatment (Rotating Biological Contactors), the required energy for treatment, pond, and irrigation is 0.8, 0.19, and 0.19 kWh·m^−3^, respectively. The energy requirement for the model's application is approximately 112 GWh·year^−1^ with treatment and storage combined, and irrigation requiring 95 and 18 GWh·year^−1^, respectively (Figure 5d). Regarding the energy harvesting potential from FPV units, the generated solar energy surpasses this requirement, with an estimated surplus of 1683 GWh·year^−1^ energy, enhancing energy security in the basin.

## 4 Discussion

Given the multidimensional nature of WEF Nexus proposals, this section discusses the feasibility of the proposed model, the environmental risks, issues of social equity and benefit distribution, climate‐related implications, and the scalability and adaptability of the approach.

### 4.1 Techno‐Economic and Operational Feasibility

First, from a technical perspective, FPV is an emerging but mature and commercially available RET. Contrary to engineering challenges associated with uncertainties in large water bodies (e.g., bathymetry, wind, and snow loads), shallow artificial water bodies such as irrigation ponds are stable and safe settings for deployment. Recent analysis of climatic conditions demonstrated that Atoyac River basin do not imply significant challenges to FPV pontoons [57]. The same study cast light on the economic challenges of FPV systems. The governmental reluctance to RETs has discouraged the private sector's wide engagement with photovoltaic technology, and this has increased the associated overhead costs of PV projects. As a result, Mexico is among the top expensive countries in PV technology [21] and a limited number of new projects have been commenced in the past presidential period [58]. This fact may be discouraging for investors, but authorities could still be persuaded by its multifaceted and national‐interest benefits in addressing inefficiencies in WEF sectors nationwide.

In a larger context, electricity prices also play a critical role. In this study, we adopted a conservative approach by using the average household electricity tariff [49]. However, given the large surplus of generation capacity, the wastewater treatment process, instead of being an energy sink and a burden on the national grid, could supply clean electricity locally to industrial users. It is important to note that, due to the lack of detailed data on the share of electricity within WWTP's OPEX function [50], the methodology is limited in demonstrating the benefits of substituting the grid's energy price from the OPEX.

Ultimately, the results indicate that, under the applied assumptions, around 35% of the generated sewage could be recycled. It is worth noting that this value is obtained by aggregating municipal‐level results based on the determining model inputs; therefore, the percentage itself should not be interpreted as a standalone factor. Upon the availability of finer data for settlements, the model can be more precisely tailored, and the resulting circulation rate may differ. For instance, small and individual settlements such as Santa Ana Nopalucan currently could exhibit higher accuracy due to the proximity of human settlement and farmland, whereas in cases such as the Puebla metropolitan area, this distance can become a limiting factor. To minimize the effect of this constraint, we assumed a small‐scale, decentralized scheme for sewage generation and treatment (2 000 p.e.) and accounted for the energy required to pump treated effluent from ponds to farms.

### 4.2 Environmental Risks

From an environmental perspective, the main risks can be grouped into two categories, those related to the installation of FPV units and those associated with the use of treated sewage for irrigation. The FPV canopy reduces light penetration into the water column, which can affect the biological life cycle in the host water body. The magnitude of this effect depends strongly on the size of the water body and the proportion of the surface covered by FPV [59]. Since the methodology presented focuses on small, artificial irrigation ponds that are not designed or managed as ecological habitats, potential impacts on the water's physicochemical properties (e.g., dissolved oxygen) are expected to be limited.

The second concern is related to the large‐scale use of treated sewage for irrigation. To comply with safety regulations, the technological assessment of available technologies has been carried out using latest national [34] and international [33] standards (Table S2) to meet hazardous thresholds. It is also worthwhile considering the ongoing pollutant fate in the basin. Currently, the Atoyac River is already highly contaminated, flows through densely populated urban areas, and has caused substantial environmental damage in the region. River flow and infiltration into aquifers have mobilized various contaminants into drinking‐water wells and contributed to an increase in disease incidence in the area [9, 60]. Moreover, limited monitoring and unrestricted access to sewage channels have encouraged informal and unregulated abstractions for irrigation, which have increased human exposure to pathogens. Taking all this into account, the presented approach improves both the safety and the effectiveness of wastewater recovery for irrigation.

### 4.3 Climate‐Related Implications

Extreme climatic events can challenge agriculture and water systems. Droughts and upward temperature anomalies increase crop evapotranspiration and irrigation water demand, while simultaneously exacerbating water scarcity. This implies higher irrigation requirements and lower sewage availability, both of which are against the model's performance; however, the most compelling comparison is with current practices. As shown in Figure 4, corn, as the staple crop, is highly vulnerable to water stress, and the current rainfed regime is highly fragile [61]. In addition, in many parts of Mexico, including the Atoyac River basin, climate change has already reduced precipitation [62, 63]. Thus, by implementing irrigation ponds, storage capacity can be used not only for storing treated sewage but also for harvesting rainwater, which can still help alleviate growing water stress.

Increased rainfall events can challenge WWTPs by raising influent volumes and, consequently, the energy consumption and associated treatment costs [64]. To address this, sewer dredging or bypass (in the worst case) strategies are often implemented [65]. However, given the decreasing precipitation trends and the high land infiltration capacity in rural and suburban areas, it is unlikely that heavy rainfall will pose significant challenges to the implementation of the model.

### 4.4 Scalability, Social Equity, and Benefit Distribution

The socio‐political sustainability of every proposal is crucial for successful implementation. The policies for water, food, and energy are currently implemented in silos, causing negative consequences for the sustainability of these sectors; therefore, we acknowledge that this proposal, at first, can face several governance challenges. To address this, our analysis has focused on quantifying the multidimensional benefits related to each one of the systems and minimizing the conflicts with ongoing practices.

Improving the water's economic productivity is critical, but water prices have been kept at an affordable level. The State Congress regulates the pricing, and governmental actors avoid realistic water pricing due to political consequences [5]. In this regard, over 90% of proposed projects are rejected by the Ministry of Finance due to the rigid water pricing policies [66]. To address this, instead of monetizing the water itself, the treatment process is monetized, enabled by recent technological developments in PV. Additionally, through careful resource management, the treated wastewater is stored for agricultural use according to the crop's growth profile.

By quantifying the projected crop yields and energy exports to the grid, the win–win nature of the model is demonstrated. Particularly, growing concerns about Mexico's heavy reliance on imported grains [67] and escalating tensions with corn farmers. Recently, the government has agreed to guarantee purchase prices that are 25% higher than the global average for domestically produced corn grain to compensate for low economic productivity [68]. At the same time, in a context where private‐sector engagement with clean energy technologies is being discouraged [57], the PV‐generated electricity can help alleviate the burden of rising energy demand from households and wastewater treatment. All in all, developing pilot projects can be an effective way to engage authorities through peer learning. These projects would validate and demonstrate the results, including the possibility of generating and exporting power to the grid and increasing corn farm yields through adequate irrigation water management.

For a just transition, equitable distribution of resources and benefits is essential. In Mexico, water is regulated by the National Water Law (Ley de Aguas Nacionales), under which CONAGUA (Comisión Nacional del Agua) and the basin agencies grant water concessions for agricultural users. In irrigated areas, concessions are often held by ejidatarios and other smallholders, which are managed through local water user associations and committees. The law allows the reuse of treated wastewater upon its quality compliance with the NOM (Official Mexican Norms) standards. The users must pay self‑sufficiency fees that at least cover the administration and OPEX of irrigation infrastructure [69]. Under this regulatory framework and local supervision system, water stored in irrigation ponds can be formally incorporated and allocated to users. Particularly, given the self‐sufficiency fees, costs related to pumping energy and pond maintenance can be compensated by users, which, in our assumptions, are conservatively aggregated into the OPEX.

## 5 Conclusions

Deficient funding for WWTP operating expenses has resulted in abandoned and underperforming treatment facilities in the Atoyac River Basin, Mexico, leading to severe environmental degradation. Additionally, agricultural practices are inefficient in many regions due to the predominance of rainfed corn farming. To inclusively address these problems, this study develops a PV‑powered circular model that independently generates income from the wastewater treatment process and uses wastewater resources for irrigation expansion.

The analysis shows that FPV technology can supply local communities with clean energy while preserving treated wastewater for the peak irrigation demand period. The storage capacity of irrigation ponds can amplify relatively small wastewater streams and enable their effective use in irrigation, thereby increasing corn yields and farmers’ revenues. These results demonstrate the potential to tackle food security, rural underdevelopment, and environmental degradation caused by wastewater discharges through a comprehensive circular economy model. Given national priorities and the techno‑economic viability of the approach, the federal government could benefit from adopting such an interdisciplinary strategy to address these challenges. Beyond Mexico, the method could be tailored to other countries with similar conditions.

### 5.1 Limitations and Perspectives for Future Research

The methodology developed has limitations. The case study is defined at a large, watershed scale, whereas the model requires localized analysis. Because of data constraints at the municipal level, this study could not be further downscaled. Furthermore, we adopted a greenfield approach for WWTPs, although available infrastructures could enhance the feasibility of the proposal. For future work, narrowing the analysis to an individual community or a small cluster of settlements could more accurately demonstrate the costs and benefits of the model. The in‐situ use of surplus power through battery storage or Power‐to‐X approaches could further enrich energy self‐sufficiency in WWTP management.

## Funding

The research was co‐founded by CAPEX: Renewable Energies Lab 25 and OPEX EIC‐ PHFT070‐22ZZ00003 at Tecnológico de Monterrey, Puebla. The research was funded by Secretaría de Ciencia, Humanidades, Tecnología e Innovación (Secihti) grant number PEE‐2025‐G‐203 under Proyectos de Investigación Científica y Humanística en Ejes Estratégicos 2025 Scholarship of Shahin Rasooli, SECIHTI grant No. 2022‐000002‐01NACF‐08059.

## Conflicts of Interest

The authors declare no conflict of interest.

## Supporting information




**Supporting File 1**: gch270082‐sup‐0001‐SuppMat.docx.

**Supporting File 1**: gch270082‐sup‐0002‐DataFile.zip

## Data Availability

The data that support the findings of this study are available from the corresponding author upon reasonable request.
