# Supplementary material for: Synergetic Benefits of Agricultural Sewage Reuse and Floating Photovoltaics in Mexican Wastewater System: A Municipal‐Level WEF Nexus Study
Source: Glob Chall. 2026 Jan 14;10(1):e00440. doi: 10.1002/gch2.202500440 (PMC12802559; doi:10.1002/gch2.202500440)
Supplement: Supplementary file 1 — Supporting File 1: gch270082‐sup‐0001‐SuppMat.docx. [file GCH2-10-e00440-s002.docx]

**Synergetic Benefits of Agricultural Sewage Reuse and Floating Photovoltaics in Mexican Wastewater System: A Municipal-Level WEF Nexus Study**

Shahin Rasooli^a^, César Casiano-Flores^b^, Shahrzad Farhoodi^a^, Bassel Daher^c^, Pabel Antonio Cervantes-Avilés^a^, Carlos Alberto Huerta-Aguilar^a*^^[[1]](#footnote-2)^

1. School of Engineering and Sciences, Tecnológico de Monterrey, 72456, Puebla, Mexico
2. Section of Governance and Technology for Sustainability, University of Twente, 7522 NH, Enschede, Netherlands
3. Department of Biological and Agricultural Engineering, Texas A&M University, 77845, Texas, USA

* Corresponding author

Email address: [huertaa@tec.mx](mailto:huertaa@tec.mx)

1. **Crop Planting Calendar**

**Supplementary Table 1.** Crop planting calendar for basin agricultural products and utilized FAO crop model.

| FAO Crop Model | Planting Date | Included Crop Types from SIAP |
| --- | --- | --- |
| Small Vegetables | April 15 | Coriander, lettuce, nopalitos, chard, Chenopodium nuttalliae, parsley, asparagus, radish, spinach, Mexican tea, garlic, carrot, onion |
| Alfalfa-perennial | Perennial-April 1 | Alfalfa, pastures |
| Barley | April 15 | Barley, barley forage |
| Sugar beet | April 15 | Sugar beet |
| Cabbage | April 15 | Cabbage, brussels sprouts, artichoke, cauliflower, broccoli |
| Sweet Melon | May 24 | Pumpkin, zucchini |
| Green Beans | June 1 | Garden pea, green fava bean, green bean, pea |
| Dry Beans | April 15 | Fava dry bean, dry bean |
| Pepper | May 24 | Chile |
| Maize | June 1 | Corn grain, forage green sorghum |
| Tomato | June 1 | Tomato, green tomato |
| Wheat | April 15 | Amarando, wheat, triticale |
| Potato | April 15 | Potato |

1. **Sewage Characteristics and Treatment Performance**

**Supplementary Table 2.** Typical composition of wastewater in wastewater treatment plants^1,2^.

| Parameter | Moderate Loaded | Treated Sewage for Irrigation  (FAO) | Irrigation Water Quality  (NOM-001-SEMARNAT-2021) | Removal  (%) |
| --- | --- | --- | --- | --- |
| Population equivalent (p.e) | 1,500 | - | - | - |
| Flow Rate (m^3^/day) | 400 | - | - | - |
| Biological Oxygen Demand (mg/l) | 500 | 50 | NA | 90 |
| Chemical Oxygen Demand (mg/l) | 1300 | 120 | 150 | 90> |
| Suspended Solids (mg/l) | 700 | 30 | 100 | 85> |
| Phosphorous (mg/l) | 20 | 10 | NA | 50 |
| Nitrogen (mg/l) | 40 | 30 | NA | 25 |

1. **Irrigation water demand modelling**


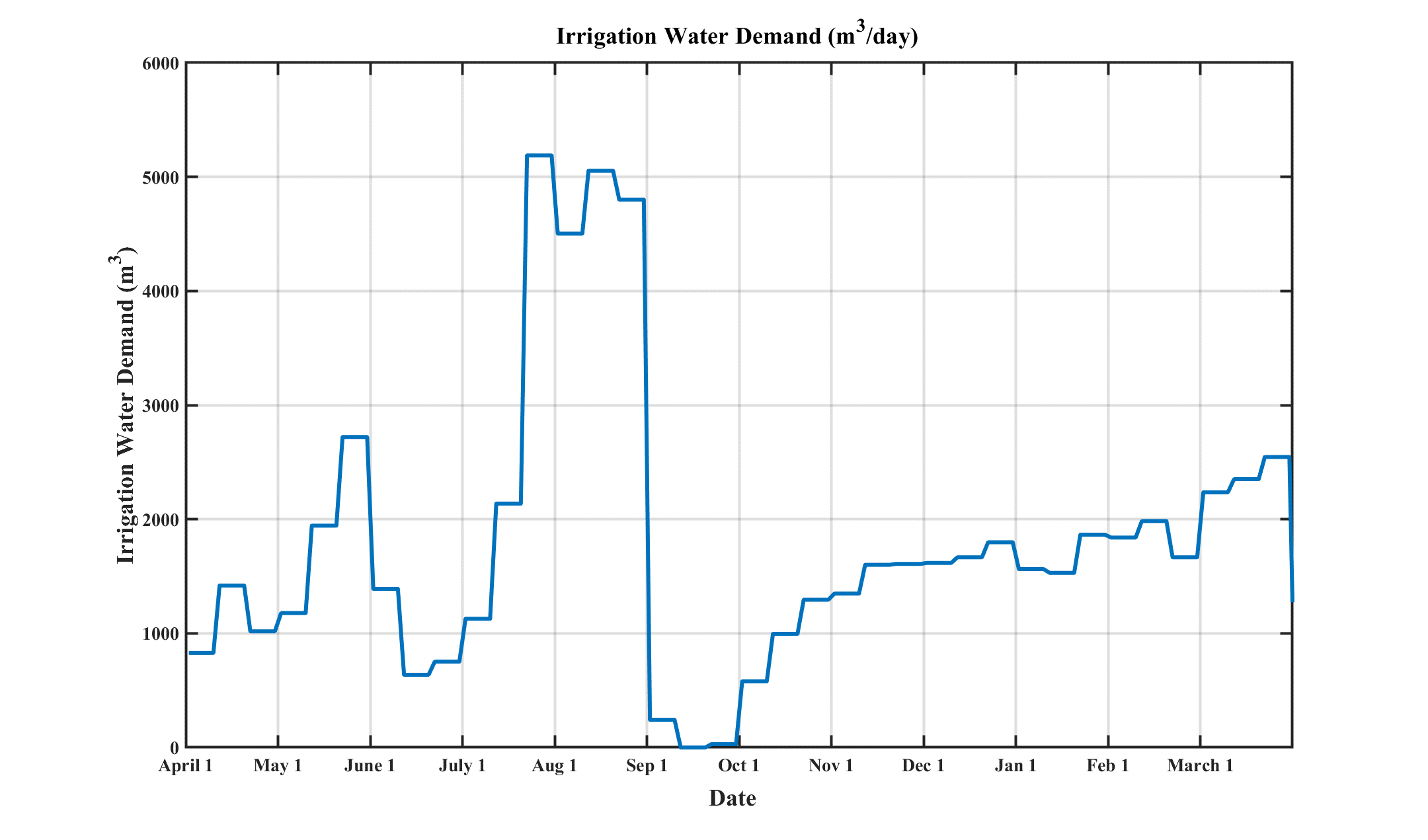


**Supplementary Figure 1.** Irrigation water demand, transformed via the Heaviside function (April 1 is starting point).

1. **FPV System Configuration**

**Supplementary Table 3.** Solar Module Specifications.

|  | Specification | Description |
| --- | --- | --- |
| Panel tilt | 23° | Average Optimum Values retrieved from PVGIS for the basin |
| Panel azimuth | 159° (South 180°) |  |
| Pmax | 550 W | ET-M772BH550WW/WB 550W |
| Module Efficiency | 21.5% |  |
| Pmax Temperature Coefficient | -0.340 %/°C |  |
| Operation period | 25 years |  |
| Dimensions | 2274 × 1134 × 35 mm | Horizontal Installation |

**
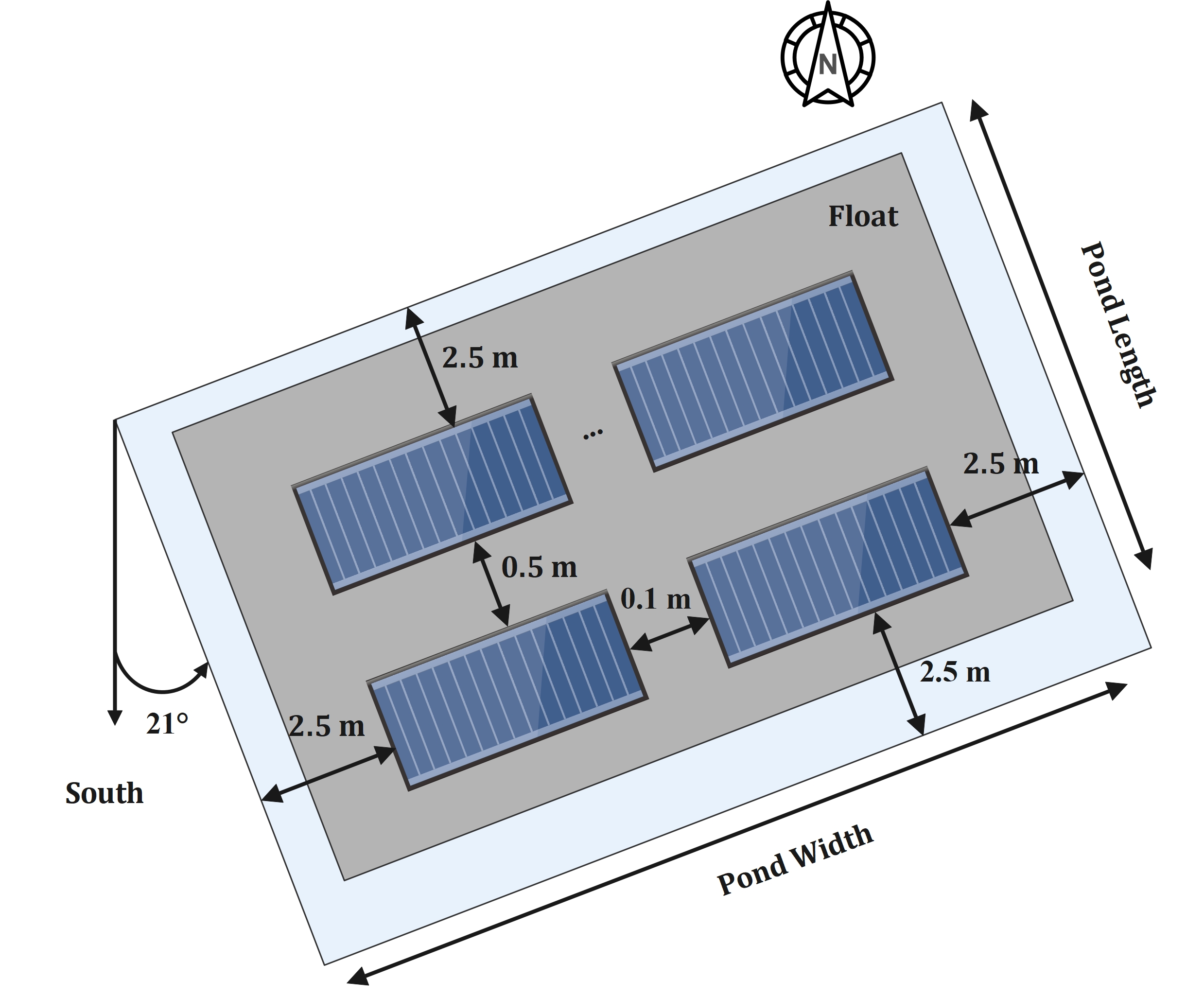
**

**Supplementary Figure 2.** Schematics of FPV system's installation.

1. **Techno-Economic Parameters**

**Supplementary Table 4.** The techno-economic factors of applied secondary treatment technologies for sewage treatment^2,3^.

| Secondary Technology | Definition | Energy  Consumption  (kWh/m^3^) | Contaminants Removal  (%) | Associated Costs  (€/p.e.) |
| --- | --- | --- | --- | --- |
| Pond System  (PS) | Artificial man-made lagoons in which wastewater is treated by natural occurring processes and the influence of solar light, wind, microorganisms, and algae. | 0.19 | N: 20-40  P: 60-70  COD: 60-96  SS: 50-90 | O&MC: y =5.543x+3,127.5  (R^2^=0.991) |
| Trickling Filter  (TF) | A fixed bed over which sewage flows downward developing a layer of microbial slime (biofilm), covering the bed of media. | 0.3 | N: 35-50  P: 35-55  COD: 75-90  SS: 50-90 | O&MC: y =13.504x+6,030.0  (R^2^=0.998) |
| Rotating Biological Contactors  (RBC) | Large disk with radial and concentric passages slowly rotating. The alternate exposure to oxygen/sewage promotes the development of a thin layer of biomass. | 0.8 | N: 20-80  P: 10-30  COD: 70-93  SS: 75-98 | O&MC: y = 313.4x^-0.435^  (R^2^=0.994) |
| Membrane Bioreactor  (MBR) | Combination of the conventional activated sludge process with a membrane filtration step. | 0.8 | N: 50-90  P: 20-70  COD: 70-90  SS: 85-99 | O&MC: y =30.150x+13,542  (R^2^=0.985) |
| Sequencing Batch Reactor  (SBR) | Fill-and-draw activated sludge system where all the operations (fill, react, settle, and draw) are achieved in a single batch reactor. | 1.0 | N: 55-90  P: 25-70  COD: 70-90  SS: 85-99 | O&MC: y =309.4x^-0.389^  (R^2^=0.950) |

1. **Cost Updating**
2. **Cost Updating for FPV Systems**

**Supplementary Table 5.** Cost breakdown and details of FPV systems^4^ and corresponding cost updating factors for FPV system components^5^.

| **Cost Breakdowns**  **USD/Wdc** | **2 MWdc** | **Description** |
| --- | --- | --- |
| Module | 0.07 |  |
| Inverter | 0.07 × 2.5 | Replacement after 10 years (× 2.5) and considered at the first year’s CAPEX |
| Structural Balance of System (SBOS) | 0.47 | × 195 (South America Q4 2022) ÷ 184 (Global Q4 2020) |
| Electrical Balance of System (EBOS) | 0.15 | × 203 (South America Q4 2022) ÷ 185 (Global Q4 2020) |
| Equipment and Installation Labor | 0.07 | × 180 (South America Q4 2022) ÷ 225 (Global Q4 2020) |
| Permitting, Inspection, Interconnection | 0.15 |  |
| Sales Tax | 0.05 |  |
| EPC OH&P | 0.10 |  |
| Shipping and Handling | 0.02 |  |
| Contingency | 0.06 |  |
| Developer Overhead | 0.14 |  |
| Profit | 0.10 |  |
| **Total** | **1.58** |  |
| **Operation and Maintenance** | **0.019** |  |

1. **Cost updating for WWTPs**

| $PCF=\frac{\mathrm{USD}}{\mathrm{Euro}}2011\cdot\frac{USGC LF}{Germany LF} 2011\cdot\frac{PCI Q4 2022}{PCI 2011}\cdot\frac{SA LF}{USGC LF}Q4 2022$ | (1) |
| --- | --- |
| $LF=CL\cdot L + EQ\cdot Eq+EPM\cdot Eng +ST\cdot St+EI\cdot Ins+ CCE\cdot CC$ | (2) |

Where LF is location factor; CL stands for Construction Labor cost index; EQ is equipment procurement cost index; EPM is engineering and project management cost index; ST is steel cost index; EI is electrical and instrumentation procurement cost index; CCE is Construction and Civil Engineering cost; Shares of Labor (L), Equipment (Eq), Engineering (Eng), Steel (St), Electrical Instruments (EI), and Construction Cost (CC) from final projects are also considered. The values for equations (1) and (2) are reported in **Supplementary Table 6** and **Supplementary Table 7**.

**Supplementary Table 6.** Exchange rate, location factor, and cost updating factors^5^.

| Factor | Year | Value |
| --- | --- | --- |
| $\frac{USD}{Euro}$ Average Exchange Rate | 2011 | 1.39 |
| Germany Location Factor | 2011 | 1.10 |
| The US Gulf Coast (USGC) Location Factor | 2011 | 1,151 |
| USGC Location Factor | Q4 2022 | 1,568 |

**Supplementary Table 7.** Project cost breakdown structure, and location factors for the fourth quarter of 2022^5^.

| Breakdown | Cost Breakdown Share  [%] | South America Location Factor | North America/USGC Location Factor |
| --- | --- | --- | --- |
| Labor | 19.8 | 180 | 327 |
| Equipment | 40.9 | 260 | 278 |
| Steel and Pipe | 15.5 | 389 | 451 |
| Engineering and Project Management | 10.6 | 165 | 292 |
| Instrumentation and Electrical | 7.7 | 203 | 233 |
| Construction and Civil (CL) | 5.5 | 195 | 224 |

**References**

1 Ayers, R. S. & Westcot, D. W. *Water quality for agriculture*. Vol. 29 (Food and agriculture organization of the United Nations Rome, 1985).

2 Molinos-Senante, M., Garrido-Baserba, M., Reif, R., Hernandez-Sancho, F. & Poch, M. Assessment of wastewater treatment plant design for small communities: environmental and economic aspects. *Sci Total Environ* **427-428**, 11-18, doi:10.1016/j.scitotenv.2012.04.023 (2012).

3 Tchobanoglus, G., Burton, F. & Stensel, H. D. Wastewater engineering: treatment and reuse. *American Water Works Association. Journal* **95**, 201 (2003).

4 Ramasamy, V. & Margolis, R. Floating photovoltaic system cost benchmark: Q1 2021 installations on artificial water bodies. (National Renewable Energy Lab.(NREL), Golden, CO (United States), 2021).

5 Kumar, G. PEP Cost Index Update. 8-15 (S&P Global, USA, 2023).

1. **Permanent Address:** Prof. Carlos Alberto Huerta-Aguilar. Laboratorio de Energias Renovables, 5718 Atlixcayotl Ave., 72453, Puebla, Mexico. [↑](#footnote-ref-2)
